# Use of artificial intelligence for public health surveillance: a case study to develop a machine Learning-algorithm to estimate the incidence of diabetes mellitus in France

**DOI:** 10.1186/s13690-021-00687-0

**Published:** 2021-09-22

**Authors:** Romana Haneef, Sofiane Kab, Rok Hrzic, Sonsoles Fuentes, Sandrine Fosse-Edorh, Emmanuel Cosson, Anne Gallay

**Affiliations:** 1 Department of Non-Communicable Diseases and Injuries, Santé Publique France, 12 rue du Val d’Onse, 94415 Saint-Maurice, France; 2 Population-Based Epidemiological Cohorts Unit, INSERM UMS 011, Villejuif, France; 3 Department of International Health, Care and Public Health Research Institute – CAPHRI, University of Maastricht University, Maastricht, The Netherlands; 4 Department of Endocrinology-Diabetology-Nutrition, AP-HP, Avicenne Hospital, Paris 13 University, Sorbonne Paris Cité, CRNH-IdF, CINFO, Bobigny, France; 5 Sorbonne Paris Cité, UMR U1153 Inserm/U1125 Inra/Cnam/Université Paris 13, Bobigny, France

**Keywords:** Artificial intelligence, Machine learning technique, Supervise learning, Health indicator, Incidence, Diabetes mellitus, Electronic health records and public health surveillance

## Abstract

**Background:**

The use of machine learning techniques is increasing in healthcare which allows to estimate and predict health outcomes from large administrative data sets more efficiently. The main objective of this study was to develop a generic machine learning (ML) algorithm to estimate the incidence of diabetes based on the number of reimbursements over the last 2 years.

**Methods:**

We selected a final data set from a population-based epidemiological cohort (i.e., CONSTANCES) linked with French National Health Database (i.e., SNDS). To develop this algorithm, we adopted a supervised ML approach. Following steps were performed: i. selection of final data set, ii. target definition, iii. Coding variables for a given window of time, iv. split final data into training and test data sets, v. variables selection, vi. training model, vii. Validation of model with test data set and viii. Selection of the model. We used the area under the receiver operating characteristic curve (AUC) to select the best algorithm.

**Results:**

The final data set used to develop the algorithm included 44,659 participants from CONSTANCES. Out of 3468 variables from SNDS linked to CONSTANCES cohort were coded, 23 variables were selected to train different algorithms. The final algorithm to estimate the incidence of diabetes was a Linear Discriminant Analysis model based on number of reimbursements of selected variables related to biological tests, drugs, medical acts and hospitalization without a procedure over the last 2 years. This algorithm has a sensitivity of 62%, a specificity of 67% and an accuracy of 67% [95% CI: 0.66–0.68].

**Conclusions:**

Supervised ML is an innovative tool for the development of new methods to exploit large health administrative databases. In context of InfAct project, we have developed and applied the first time a generic ML-algorithm to estimate the incidence of diabetes for public health surveillance. The ML-algorithm we have developed, has a moderate performance. The next step is to apply this algorithm on SNDS to estimate the incidence of type 2 diabetes cases. More research is needed to apply various MLTs to estimate the incidence of various health conditions.

**Supplementary Information:**

The online version contains supplementary material available at 10.1186/s13690-021-00687-0.

## Background

The availability of administrative data generated from different sources is increasing and the possibility to link these data sources with other databases offers unique opportunity to answer those research questions, which require a large sample size or detailed data on hard-to-reach population [1]. French National Health Data System (i.e., SNDS [*Système National de Données Santé]*) is an example of a big data/large administrative linked data set, which is used for public health surveillance in France [2]. It includes most updated, individual level health information about health insurance claims, hospital discharge and mortality of whole French population (i.e., 66 million people) [2]. However, the estimation of health indicators from linked administrative data is challenging due to several reasons such as variability in data sources and data collection methods, availability of a large number of variables, lack of skills and capacity to analyze big data [3]. More efficient ways of analyzing health information using big data across European countries are required. In that context, the use of artificial intelligence (AI) is increasing in healthcare. Indeed AI allows to handle data with a large number of dimensions (features) and units (feature vectors) efficiently with a high precision. AI techniques offer benefits in estimation of health indicators both at individual and population levels (i.e., improving social and health policy process). Machine learning (ML) is an application of AI that provides systems the ability to learn automatically and improve from experience without being explicitly programmed [4]. Supervised learning algorithms build on a mathematical model of a set of data that contains both the inputs and the desired outputs [5]. This approach is based on the prior knowledge of what the output values for a given sample should be [6]. ML techniques have been applied for the diagnosis of certain conditions as well as outcome prediction and prognosis evaluation with high precision [7–9].

This study was carried out under the InfAct (Information for Action) project [10], which is a joint action of Member States aiming to develop a more sustainable European health information system through improving the availability of comparable, robust and policy-relevant health status data and health system performance information. InfAct gathers 40 national health authorities from 28 Member States. This study is part of a work package (WP9) focused on innovation in health information system (i.e., using data linkages and/or AI) to improve public health surveillance and health system performance for health policy process. As a first step, we have explored the current usage of these innovative techniques (i.e., data linkages and/or AI) in European countries and very few countries apply AI to estimate health indicators in their public health activities [11]. Therefore, the next step was to develop a generic approach by applying these innovative techniques to estimate the health indicators of chronic conditions for improved surveillance.

We used diabetes as a case study due to several reasons. First, it is one of the leading cause of morbidity in the world [12] and its prevalence is increasing among all ages in the European region, mostly due to increase in overweight and obesity, unhealthy diet and physical inactivity [13]. Second, a training data set using CONSTANCES cohort was already developed and used to answer various research questions for diabetes. Third, as this study is part of the InfAct project with a limited period to be completed. Fourth, estimation of incidence of diabetes cases is important to develop the prevention strategies to reduce its burden. For example, promoting healthy diet and physical activity in daily life could reduce the risk of developing diabetes 2.

The main objective of this study was to develop for the first time a generic ML-algorithm to estimate the incidence of diabetes based on the number of reimbursements over the last 2 years, excluding the anti-diabetes drugs as predictors and focused on non-diabetic participants over the last 2 years.

## Method

### Development of the ML-algorithm

To develop ML-algorithm, we adopted a supervised ML approach using R-software (R × 64 3.6.3) and used following key libraries: caret 6.0–86, AppliedPredictiveModeling 1.1–7, CORElearn 1.54.2, C50 0.1.3.1, and xgboost 1.3.2.1. Following steps were performed: i. selection of final data set, ii. target definition, iii. Coding of variables for a given window of time, iv. split final data into training and test data sets, v. variables selection, vi. training model, vii. Validation of model with test data set and viii. Selection of the model.

#### i. Selection of final data set

We selected a final data set from a population-based epidemiological cohort (i.e., CONSTANCES) to develop an algorithm to estimate the incidence of diabetes. The participants were recruited by CONSTANCES between January 1, 2012, and December 31, 2014. This cohort comprises after final completion a national representative randomly selected sample of 50,954 aged between 18 and 69 years (inclusive) and living in France [14, 15]. The participants are randomly selected from the beneficiaries of the National Health Insurance Fund (i.e. CNAM [Caisse Nationale d’Assurance Maladie]). In this cohort, data are collected using a self-administered questionnaire (SAQ) and a medical questionnaire (MQ) and were used to define the known diabetes cases and pharmacologically-treated diabetes [16]. For known diabetes cases, in the SAQ, participants reported to have diabetes through the item: *“Have you ever been told by a doctor or other health care professional that you had diabetes?”* In the medical questionnaire, completed during the medical examination, the physician asked each participant if they had diabetes. For the pharmacologically-treated diabetes, two questions in the medical questionnaire were related to diabetes treatment: “*Are you currently being treated for diabetes with oral medication?”* And *“Are you currently being treated for diabetes with one or more insulin injections?”* [16]*.*

After fulfilling a SAQ on health status, life style factors, socioeconomic and demographic characteristics, the participants attend to their related health screening center for a medical examination which includes: medical questionnaire, physical examination and blood sampling. This information previously collected was linked with the French National Health Data System (i.e., SNDS). We excluded women who declared being already diagnosed of gestational diabetes mellitus, pregnant women, no data on participants in SNDS, incomplete data in SAQ/MQ, incomplete data on age of diabetes diagnosis, diabetes cases who were declared before 12 months of SAQ/MQ and all participants with antidiabetic drug reimbursement between 12 and 36 months before SAQ/MQ. Moreover, we considered gestational diabetes as a special group and excluded these women due to their different physiopathology. Some may develop the diabetes earlier or later due to hormonal disturbance. To predict the incidence of diabetes among these women required specific case definitions.

#### ii. Target definition

The target definition includes the participants who declared diabetes in CONSTANCES cohort (first occurrence ≤12 months) with the first antidiabetic drug reimbursement between 0 and 12 months before inclusion. The diabetes status at inclusion (M0: inclusion in CONSTANCES) was defined according to CONSTANCES as described above. The linkage with the French National Health Data System (i.e., SNDS) allowed recording all antidiabetic drug reimbursement between 0 and 36 months before inclusion (SAQ/MQ).

**Fig. 1:**
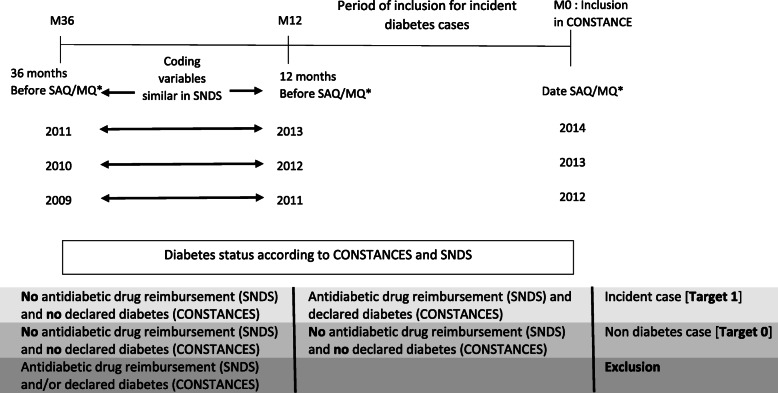
Target definition in CONSTANCES Cohort, “A case study performed in 2019-20 to develop a Machine Learning-algorithm to estimate the incidence of Diabetes Mellitus in France”. *SAQ: Self-administered Questionnaire. MQ: Medical Questionnaire

Participants without declared diabetes (CONSTANCES SAQ/MQ) and/or antidiabetic drug reimbursement between 0 and 36 months before inclusion were defined as non-diabetes cases (target 0). Participants who declared diabetes in CONSTANCES SAQ/MQ (first occurrence ≤12 months) with the first antidiabetic drug reimbursement ≤12 months before inclusion were defined as incident cases (target 1). These diabetes cases included both type 1 and 2. Participants with antidiabetic drug reimbursement between 12 and 36 months before inclusion or declared diabetes (first occurrence > 12 months) were excluded (see Fig. 1). We excluded these participants to avoid the potential influence of anti-diabetes drugs on the estimation of incidence of diabetes (not true incident cases).

#### iii. Coding of variables for a given window of time

In CONSTANCES, we only coded those variables, which were also available in the SNDS to apply the potential ML-algorithm on SNDS to estimate the incidence of diabetes. A total of 3483 continuous variables were coded and standardized using the z-score transformation (for each data point, we subtracted the mean and divided with the standard deviation) over the last 24 months before the date of SAQ. The rational to have a time window of 24 months before the SAQ was to provide a long duration to study changes in diagnostic procedures, hospitalizations and drug consumption that allows to estimate the incidence of diabetes with high accuracy. Following were the main categories of variables: number of medical consultations (50 variables), drug dispensed coded using the 5th level of the Anatomical Therapeutic code [ATC 05] (461 variables), biological test (747 variables), medical acts (i.e., X-ray, surgery, etc.) (2135 variables), all hospitalizations (5 variables), hospitalizations with a procedure (i.e., dialysis, radiotherapy, etc.) (5 variables), hospitalizations without a procedure (5 variables), hospitalizations related to following associated health conditions: diabetes, heart failure, stroke, heart attack, foot ulcer, lower limb amputation, ischemic heart disease, transient ischemic attack, end-stage renal failure, diabetic coma, diabetic ketoacidosis and cancer (75 variables).

#### iv. Split final data set into training and test data sets

The final data set was randomly split into 80% as a training data set and 20% as test data set. There was a significant imbalance of number of positive target (i.e., target 1 = diabetes treated cases) over the number of negative target (i.e., target 0 = non-diabetes cases) in the training dataset. To avoid the bias in ML-algorithm and skew in class distribution, we performed a random down sampling in the training data set in target 0 group to achieve the same number of individuals in both target groups. This includes 35,728 participants where target 0 includes 35,663 participants and target 1 includes 65. Random down sampling was performed on target 0 until to achieve the same number of target 1 i.e., 65.

The selection of variables and the model was performed using the training data. The test data was used solely to test the final model performance.

#### v. Variables selection

First, we removed all variables with a variance equal to zero and then the ReliefF exp. score was estimated, based on the relevance of each variable, to differentiate between target 1 and target 0. The ReliefF expRank method is noise tolerant and is not affected by features interactions [17–19]. All the variables were ranked according to the ReliefF exp. score. For continuous variables, the score values range from 0 to 1 [18]. The cutoff score was 0.01 and was selected based on the visual inspection of the ordered plot of ReliefF values for all variables, called “elbow plot” approach. The variables that had a ReliefF exp. score equal or more than 0.01 were included to train different models and the variables less than 0.01 were excluded.

#### Steps vi to viii model selection and validation of the model with test data set

The four following models [i.e., 1. Linear discriminant analysis (LDA), 2. Logistic regression (LR), 3. Flexible discriminant analysis (FDA) and 4. Decision tree model (C5)] were applied to the training data set. We also fit three boosted algorithms (1. Boosted logistic regression, 2. Boosted C5, and 3. XGBoost), to test whether these more computationally intensive algorithms can perform significantly better than the more standard four models. The AUC is the most commonly used evaluation/performance metric in machine learning studies and measure the ability of a classifier to distinguish between classes. We used the area under the receiver operating characteristic curve (AUC) in both the cross-validation steps and the best (final) algorithm. For each model, we compared the performance in terms of AUC. We used five-fold cross-validation repeated three times to fit each model in the training stage. After that, the models’ performances were assessed using the testing data set. We calculated the mean distributions of variables to highlight each predictor, the relative difference in the two predicted groups (diabetic, non-diabetic), for example the mean of age was 56.70 y for the predicted diabetics and 43.69 y for non-diabetics.

### Sensitivity analysis

Unbalanced training data set can skew the class distribution that may affect the performance of the machine learning algorithm. To address this issue, random resampling was applied to balance the training data set and to avoid the bias estimation. We applied two following techniques: 1. over sampling is the random repeated sampling from the minority class (positive target: diabetes cases) that artificially inflates its prevalence and 2. Down sampling is the random repeated sampling from the majority class (negative target: non-diabetes cases) that artificially reduces its prevalence. The sensitivity analysis was performed for over and down sampling approaches on the test data set. Then, we automated the model selection process by giving the computer a specific metric including sensitivity, specificity, positive predictive value, negative predictive value and kappa. Finally, a single model was retained based on its performance and its transferability to other databases.

## Results

### Final data set

**Fig. 2:**
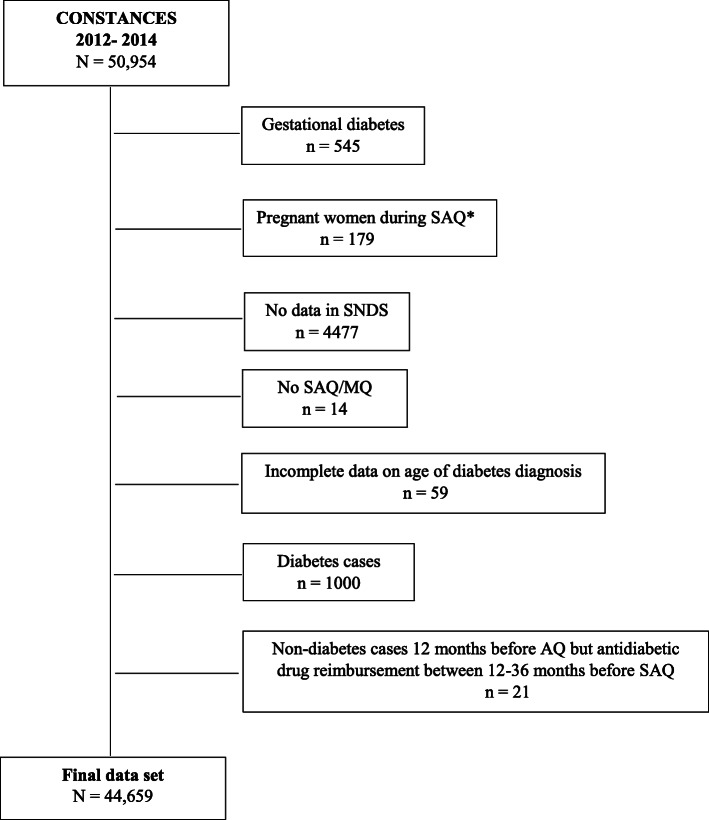
Flow chart for the selection of the final data set from CONSTANCES Cohort, “A case study performed in 2019-20 to develop a Machine Learning-algorithm to estimate the incidence of Diabetes Mellitus in France”. *SAQ = Self-administered Questionnaire. MQ = Medical Questionnaire

The final data set to develop the algorithm included 44,659 participants, with 81 incident diabetes cases (target 1) and 44,578 participants without diabetes (target 0) (Fig. 2). The general characteristics of the final data set is described in Table 1. The incident diabetes group was included older, with a higher percentage of men, treated hypertension and dyslipidemia, former smokers, a higher body mass index and a family history of diagnosed diabetes as compared to non-diabetes group.

**Table 1:** General characteristics of final data set (i.e., study population). “A case study performed in 2019-20 to develop a Machine Learning-algorithm to estimate the incidence of Diabetes Mellitus in France”

Study Population	Total (***N*** = 44,659)	***N*** = 44,659	
		Target 0 (Non-diabetes cases = 44,578)	Target 1 (Incident diabetes cases = 81)
Age in years, mean (±SD)	47.8, ±13.2	47.8, ±13.2	57.0, ±8.2
Gender, men % (n)	46.9 (20946)	46.9 (20896)	61.7 (50)
Smoking status, % (n)			
Never smoked	43.2 (19296)	43.2 (19271)	30.9 (25)
Former smoker	33.1 (14772)	33.1 (14741)	38.3 (31)
Current smoker	18.6 (8320)	18.6 (8307)	16.0 (13)
Missing	5.1 (2271)	5.1 (2259)	14.8 (12)
Body mass index, kg/m^2^ (mean, ±SD), (n)	25.0, ±4.4 (43668)	25.0, ±4.4 (43588)	31.8, ±6.0 (80)
Treated hypertension, yes, % (n)	11.3 (5031)	11.2 (4996)	43.2 (35)
Treated dyslipidemia, yes, % (n)	8.1 (3635)	8.1 (3609)	32.1 (26)
Mother/father diagnosed with diabetes, yes, % (n)	15.1 (6764)	15.1 (6730)	42.0 (34)
Education^a^ % (n)			
No education - primary education	3.1 (1374)	3.1 (1366)	9.9 (8)
Lower secondary education	6.9 (3060)	6.8 (3042)	22.2 (18)
Upper secondary education	33.5 (14942)	33.4 (14911)	38.3 (31)
Lower tertiary education	33.0 (14728)	33.0 (14714)	17.3 (14)
Upper tertiary education	21.7 (9709)	21.8 (9699)	12.3 (10)
Missing or other category	1.9 (846)	1.9 (846)	0 (.)
Geographical origin, % (n)			
Metropolitan France	87.9 (39249)	87.9 (39177)	88.9 (72)
FOT^b^	0.9 (381)	0.9 (379)	2.5 (2)
Europe	4.2 (1861)	4.2 (1859)	2.5 (2)
North Africa	2.8 (1260)	2.8 (1257)	3.7 (3)
Sub-Saharan Africa	1.1 (503)	1.1 (502)	1.2 (1)
Asia	0.7 (326)	0.7 (326)	. (.)
Others	1.0 (433)	1.0 (433)	. (.)
Missing or don’t want to answer	1.4 (646)	1.4 (645)	1.2 (1)
Professional activity, % (n)			
Employed	65.2 (29123)	65.3 (29093)	37.0 (30)
Unemployed	6.1 (2721)	6.1 (2712)	11.1 (9)
Retired	21.8 (9753)	21.8 (9720)	40.7 (33)
Student	1.5 (653)	1.5 (653)	0 (.)
Unemployed due to disability	0.9 (390)	0.9 (385)	6.2 (5)
No professional activity	1.4 (603)	1.4 (602)	1.2 (1)
Missing or other category	3.2 (1416)	3.2 (1413)	3.7 (3)

*SD* Standard Deviation

^a^ Based on the International Classification ISCED

^b^ French overseas territories

### Variables selection

**Fig. 3:**
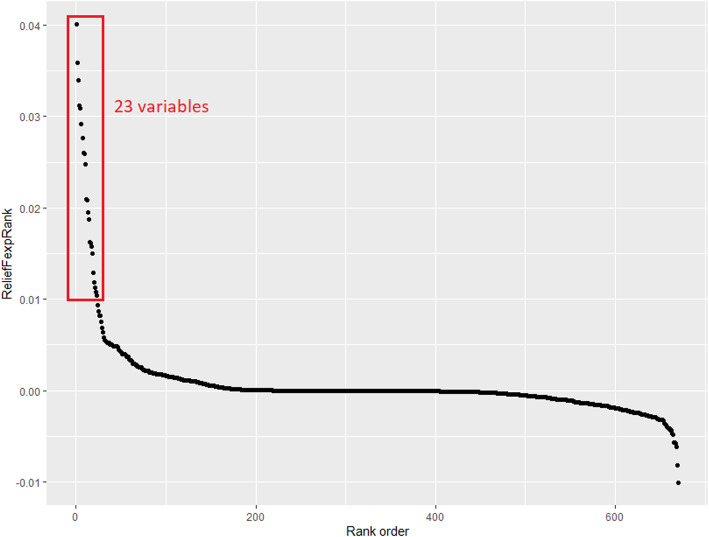
Variables selection based on ReliefF Exp Score, “A case study performed in 2019-20 to develop a Machine Learning-algorithm to estimate the incidence of Diabetes Mellitus in France”

Out of 3468 continuous variables coded, 23 variables (0.7%) had a ReliefF exp. Score above 0.01, ranked based on this score and were therefore selected (Fig. 3) (Table 2).

**Table 2:** List of selected variables ranked based on their ReliefF Exp Score. “A case study performed in 2019-20 to develop a Machine Learning-algorithm to estimate the incidence of Diabetes Mellitus in France”

Ranked #	CATEGORIES	Independent Variables
1	AGE	Age in years
**Diabetes related variables**		
6	BIOLOGICAL TESTS	Nb. of reimbursement of Glucose blood test in last 2 years
9	BIOLOGICAL TESTS	Nb. of reimbursement of HbA1c tests in last 2 years
18	MEDICAL ACTS	Nb. of reimbursement of Fundus examination by biomicroscopy with contact lens in last 2 years
19	MEDICAL ACTS	Nb. of reimbursement of functional examination of the ocular motricity in last 2 years
20	MEDICAL ACTS	Nb. of reimbursement of binocular vision examination in last 2 years
**Non-diabetes related variables**		
2	BIOLOGICAL TESTS	Nb. of reimbursement of Alkaline Phosphatase test in last 2 years
3	BIOLOGICAL TESTS	Nb. of reimbursement of Gamma Glutamyle Transferase test in last 2 years
4	BIOLOGICAL TESTS	Nb. of reimbursement of Transaminases (ALAT and ASAT, TGP and TGO) blood test in last 2 years
5	BIOLOGICAL TESTS	Nb. of reimbursement of Uric Acid (Uricemia) blood test in last 2 years
7	BIOLOGICAL TESTS	Nb. of reimbursement of Creatinine level blood test in last 2 years
8	BIOLOGICAL TESTS	Nb. of reimbursement of Exploration of a Lipid Anomaly (ELA) blood test in last 2 years
10	BIOLOGICAL TESTS	Nb. of reimbursement of C-Reactive Protein test in last 2 years
11	DRUGS	Nb. of reimbursement of Proton pump inhibitors drugs in last 2 years
12	DRUGS	Nb. of reimbursement of other antidiarrheal drugs in last 2 years
13	DRUGS	Nb. of reimbursement of Penicillin with broad spectrum drugs in last 2 years
14	DRUGS	Nb. of reimbursement of bacterial and viral vaccines, combined (diphtheria-haemophilus influenza B-pertussis-tetanus-hepatitis B-meningococcal A + C) in last 2 years
15	DRUGS	Nb. of reimbursement of Acetic acid derivatives and related substances in last 2 years
16	DRUGS	Nb. of reimbursement of Propionic acid derivatives in last 2 years
17	DRUGS	Nb. of reimbursement of Anilides (Paracetamol) in last 2 years
21	MEDICAL ACTS	Nb. of reimbursement of mammography, in last 2 years
22	MEDICAL ACTS	Nb. of reimbursement of X-ray thorax in the previous 2 years in last 2 years
23	HOSPITALIZATION	Total number of hospitalizations without a procedure (i.e. dialysis, chemotherapy) in last 2 years

The first variable was the “age”. The following nine were related to “number of reimbursements of biological tests performed in last 2 years” (i.e., Alkaline Phosphatase test, Gamma Glutamyle Transferase test, Transaminases (ALAT and ASAT, TGP and TGO) blood test, Uric Acid (Uricemia) blood test, glucose blood, Creatinine level blood test, Exploration of a Lipid Anomaly (ELA) blood test, HbA1c test and C-Reactive Protein test). The next seven were related to “number of reimbursements of various non-diabetes drugs in last 2 years” (i.e., Proton pump inhibitors drugs, antidiarrheal drugs, Penicillin with broad spectrum drugs, bacterial and viral vaccines, Acetic acid derivatives, Propionic acid derivatives and Anilides (Paracetamol). The following five were related to “number of reimbursements of various medical acts” (i.e., fundus examination by biomicroscopy with contact lens, functional examination of ocular motricity, binocular vision examination, mammography and X-ray for thorax). The last one is “the total number of hospitalizations without a procedure (i.e., dialysis, chemotherapy) in last 2 years”.

### Algorithm to estimate the incidence of diabetes

**Fig. 4:**
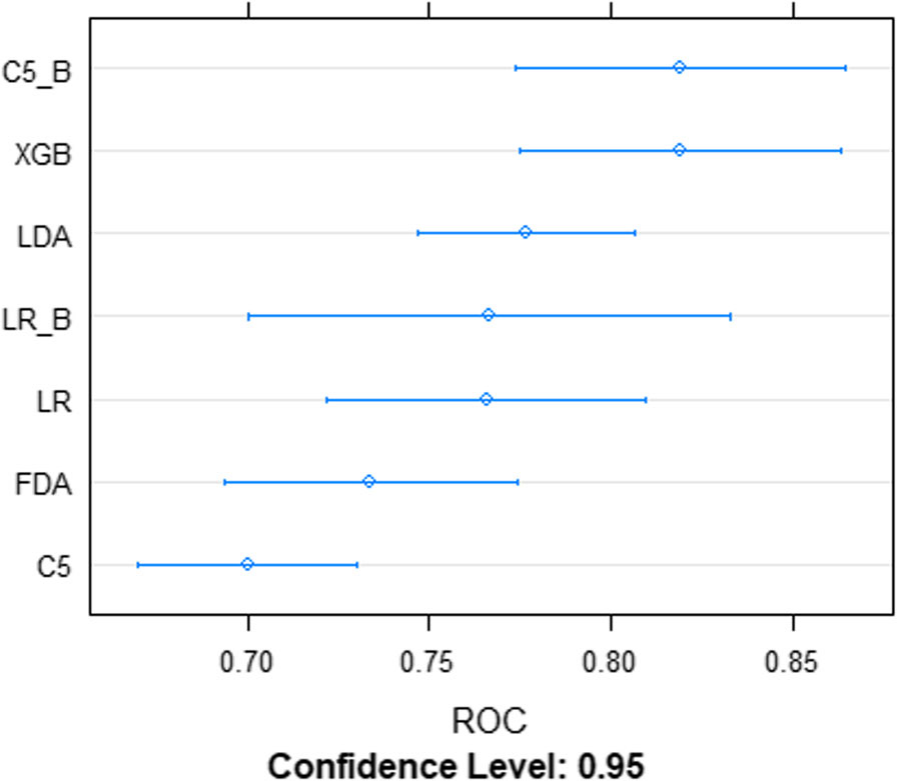
Area under the receiver operating characteristic curve and the empirical 95% confidence interval (based on fifteen resamples with replacement) for all models using the test dataset, “A case study performed in 2019-20 to develop a Machine Learning-algorithm to estimate the incidence of Diabetes Mellitus in France”

After the selection of variables, four different models [i.e., 1. Linear discriminant analysis (LDA), 2. Logistic regression (LR), 3. Flexible discriminant analysis (FDA) and 4. Decision tree model (C5)], were trained with the training dataset using three repeats of five-fold cross-validation graph. The performance of each of the model was tested on the test dataset and was measured by AUC, for which an empirical 95% confidence interval was calculated based on 15 resamples with replacement (Fig. 4). After that, we compared the performances of these four models using test data set to select the one based on the performance metrics (Table 3). We kept the LDA model since it showed a better performance with an accuracy of 67% with the test data set as compared to other models (Table 3). The three boosted algorithms improved on the predictive performance as compared to the standard models (Fig. 4). The accuracy of boosted version of logistic regression, the boosted C5.0 classification model, and XGBoost was 77, 67 and 69%, respectively (see Additional file 1). The results of sensitivity analysis are reported in the Additional file 1.

**Table 3:** Model performance evaluation with test data set, “A case study performed in 2019-20 to develop a Machine Learning-algorithm to estimate the incidence of Diabetes Mellitus in France”

	LDA	LR	FDA	C5
Accuracy	0,67	0,65	0,66	0,64
95% CI:	(0,66-0,68)	(0,64-0,66)	(0,65-0,67)	(0,63-0,65)
No Information Rate:	0,998	0,998	0,998	0,998
*P*-Value [Acc > NIR]:	1000	1000	1000	1000
Kappa	0,003	0,004	0,002	0,003
McNemar’s Test *P-*Value	<2e-16	<2e-16	<2e-16	<2e-16
Sensitivity	0,625	0,750	0,563	0,625
Specificity	0,673	0,650	0,661	0,640
Pos Pred Value	0,003	0,004	0,003	0,003
Neg Pred Value	0,999	0,999	0,999	0,999
F1-statistics	2,50	3,0	2252	2,50
Detection Rate	0,001	0,001	0,001	0,001
Balanced Accuracy	0,649	0,700	0,612	0,633

### Distribution of means of selected variables in test data set

After the selection of LDA model, the 23 selected variables were trained with the test data set (20% of final data set 44,659 = 8931). We compared the distribution of means of these continuous variables among two groups: incident diabetes cases (i.e., 2921) and non- diabetes incident cases (i.e., 6010) using LDA algorithm in the test data set (Table 4). The 2921 diabetes patients in the test dataset are the predicted cases. The mean distribution of all selected variables related to the number of reimbursements of biological tests, medicines not used for diabetes treatment and medical acts performed in last 2 years, was significantly higher in the incident diabetes group than in non-diabetes group. For example, the age was the first ranked variable with 0.04 ReliefF exp. score among 23 selected variables and was highly discriminant in the incident diabetes group. The mean age of patients in diabetes group was 57 years old as compared to 44 years old in non-diabetes group (Table 4).

**Table 4:** Distribution of means of variables selected for the final algorithm in test data set using Linear Discriminant Analysis (LDA) model, case study performed in 2019–20 to estimate the incidence of Diabetes Mellitus in France

Ranked ≠	Categories	Variables	Mean (incident diabetes group) ***n*** = 2921	Mean (non- diabetes group) ***n*** = 6010	***P*** value (Student’s t-test)
1	AGE	Age in years	56.70*	43.69	< 0.0001
2	BIOLOGICAL TESTS	Nb. of reimbursement of Alkaline Phosphatase test in last 2 years	0.36*	0.20	< 0.0001
3	BIOLOGICAL TESTS	Nb. of reimbursement of Gamma Glutamyle Transferase test in last 2 years	1.05*	0.40	< 0.0001
4	BIOLOGICAL TESTS	Nb. of reimbursement of Transaminases (ALAT and ASAT, TGP and TGO) blood test in last 2 years	1.33*	0.63	< 0.0001
5	BIOLOGICAL TESTS	Nb. Of reimbursement of Uric Acid (Uricemia) blood test in last 2 years	0.47*	0.22	< 0.0001
6	BIOLOGICAL TESTS	Nb. of reimbursement of Glucose blood test in last 2 years	1.29*	0.74	< 0.0001
7	BIOLOGICAL TESTS	Nb. Of reimbursement of Creatinine level blood test in last 2 years	1.09*	0.39	< 0.0001
8	BIOLOGICAL TESTS	Nb. of reimbursement of Exploration of a Lipid Anomaly (ELA) blood test in last 2 years	1.20*	0.65	< 0.0001
9	BIOLOGICAL TESTS	Nb. Of reimbursement of HbA1c tests in last 2 years	0.20*	0.04	< 0.0001
10	BIOLOGICAL TESTS	Nb. Of reimbursement of C-Reactive Protein test in last 2 years	1.16*	0.38	< 0.0001
11	DRUGS	Nb. Of reimbursement of Proton pump inhibitors drugs in last 2 years	3.67*	0.54	< 0.0001
12	DRUGS	Nb. Of reimbursement of other antidiarrheal drugs in last 2 years	0.21*	0.03	< 0.0001
13	DRUGS	Nb. of reimbursement of Penicillin with broad spectrum drugs in last 2 years	0.74*	0.23	< 0.0001
14	DRUGS	Nb. Of reimbursement of bacterial and viral vaccines, combined (diphtheria-haemophilus influenza B-pertussis-tetanus-hepatitis B-meningococcal A + C) in last 2 years	0.23*	0.13	< 0.0001
15	DRUGS	Nb. of reimbursement of Acetic acid derivatives and related substances in last 2 years	0.57*	0.19	< 0.0001
16	DRUGS	Nb. of reimbursement of Propionic acid derivatives in last 2 years	1.53*	1.07	< 0.0001
17	DRUGS	Nb. of reimbursement of Anilides (Paracetamol) in last 2 years	3.65*	1.69	< 0.0001
18	MEDICAL ACTS	Nb. of reimbursement of Fundus examination by biomicroscopy with contact lens in last 2 years	0.30*	0.03	< 0.0001
19	MEDICAL ACTS	Nb. of reimbursement of functional examination of the ocular motricity in last 2 years	0.19*	0.08	< 0.0001
20	MEDICAL ACTS	Nb. of reimbursement of binocular vision examination in last 2 years	0.32*	0.10	< 0.0001
21	MEDICAL ACTS	Nb. of reimbursement of mammography in last 2 years	0.12*	0.10	0.0062
22	MEDICAL ACTS	Nb. Of reimbursement of X-ray thorax in the previous 2 years in last 2 years	0.20*	0.07	< 0.0001
23	HOSPITALIZATION	Total number of hospitalizations without a procedure (i.e. dialysis, chemotherapy) in last 2 years	0.89*	0.26	< 0.0001

*Highest mean

Following the age variable, nine other features selected, related to the mean number of reimbursements of biological tests, were more discriminant (i.e., the behavior of distinguishing features or characteristics of variables by comparing two groups) in incident diabetes group than in non-diabetes group. These biological tests were performed to measure the normal values of certain enzymes, proteins, glucose and uric acid in the blood to check the normal functions of liver, kidney, pancreas and other organs. For example, the mean number of reimbursement of blood glucose test in last 2 years was 1.74 times higher in diabetes predicted group than in non-diabetes predicted group. The following group of features was the mean number of reimbursements of drugs. There were seven drugs and their mean number of reimbursements in last 2 years was more discriminant in incident diabetes group than in non-diabetes group. In the category of medical acts, there were three following features more discriminant in incident diabetes group: mean number of reimbursements of examination of fundus by biomicroscopy with contact lens, ocular motricity and binocular vision in last 2 years.

There were seven unusual features selected by the ML-algorithm and were discriminant in incident diabetes group: mean number of reimbursements of broad-spectrum penicillin, vaccines, propionic acid, Anilides (Paracetamol), mammography, X-ray for thorax and mean number of hospitalizations without any procedure.

## Discussion

We have developed an algorithm based on the supervised ML approach to estimate the incidence of diabetes using a training data set from a cohort study. This algorithm (i.e., LDA model) was built on 23 selected variables from the CONSTANCES based on the number of reimbursements over the last 2 years to estimate the incidence of diabetes. This algorithm showed a moderate performance in predicting the incidence of diabetes cases with a sensitivity of 62% and an accuracy of 67%. Among 23 selected variables, six were related to diabetes that were expected, such as age and Glucose blood test. Whereas 17 other variables were not directly related to diabetes and were more discriminant in incident diabetes group than in non-diabetes group such as Proton pump inhibitors drug.

### Main limitations of the ML-algorithm

This study was performed as a proof of concept using ML-approach to estimate the incidence of diabetes cases from reimbursement data. The results have highlighted low accuracy and there are several aspects that could explain this low accuracy. Here are some limitations: *first,* small number of diabetes-treated cases in the final data set, which could be related to the lack of older population in CONSTANCES cohort (participants between 18 and 69 years at inclusion), maybe potentially linked with low accuracy. Participation in a cohort like CONSTANCES is cumbersome and demands additional time to take part in health examinations. People in less good health and having co-morbidities (including both old and young people), require regular health check-ups therefore, they could less motivation to participate in cohort studies. Thus, it required to wait few years of follow-up for volunteers to include older age groups to have more incident diabetes cases. The risk of developing diabetes increases with age, therefore by including larger number of older people in the final dataset, the performance of this algorithm may be improved. *Second,* the time window used to code the variables was previous 2 years, was long duration. We included a longer window to better evaluate the changes in diagnostic procedures, number of hospitalizations and drug consumption and to estimate the incidence of diabetes with high accuracy. More research is needed to explore different time windows and their impact on accuracy level of estimates. *Third* is related to diabetes, which is a complex medical condition with two major clinical types of diabetes, type 1 diabetes and type 2 diabetes. The pathology and dynamics of developing these two types of diabetes are very different. The type 1 diabetes is thought to be due to autoimmunological destruction of the Langerhans Islets hosting pancreatic-ß-cells. It is diagnosed at very early stage of life and is believed to involve a combination of genetic and environmental factors. Whereas the main causes of type 2 diabetes are due to lifestyle, physical activity, dietary habits and genetic, is usually developed at later than 50 years of life. In our study, we defined the pharmacologically treated diabetes cases as target 1 and non-diabetes cases as target 0. However, we did not explicitly define the pharmacological treated diabetes cases to be further characterized as type 1 and type 2. With the inclusion of this information in the model, the accuracy level of the model could be enhanced. *Fourth,* the predictors from reimbursement data may not have a strong ability to predict the diabetes outcome. *Fifth,* these results highlight the low level of model specificity. Considering the low incidence of diabetes, it is important to take into account that insufficient model specificity could lead to the overestimation of the diabetes incidence. These results should be interpreted with caution. *Sixth,* we applied an automated procedure and selected 23 the most related variables to our outcome based on a ReliefF exp. score equal or more than 0.01. We did not consult is with a clinician. We recommend to combining both upstream clinical expertise and automated approaches for future research, for example by defining more complex indicators (more specific algorithms) related to specific conditions with higher risk of developing diabetes such as hypertension or dyslipidaemia, this can improve the performance of the algorithms. *Finally,* using the boosted algorithm for over sampling requires a high computational capacity and may take several days to compute the results. The computational capacity that is currently routinely available to public health institutions limits the attractiveness of complex analytical models that, while potentially highly accurate, may require several days to fit. This often results in such models being the product of specific research projects instead of being routinely developed in-house to support population health monitoring and clinical decision-making. This unfortunately partially limits the access to the advantages of advanced analytics. We therefore recommend that public health institutions invest in high-performance computing capacity.

Despite these limitations of this ML-algorithm, this study has some strengths: *first* is using supervised machine learning approach, we have developed the innovative methodology and could be applied to address other research questions. *Second,* this approach allows to reduce the dimension of a large number of variables (i.e., 3468) and identifies the most relevant variables (i.e., 23/3468 = 0.7%) to the desired outcomes more efficiently. The LDA model has been used for variables selection and dimensionality reduction for diabetes diagnosis [20]. *Third,* it allows to identify new variables, which were not observed using classical statistical approaches and can enrich the information to estimate the health indicators.

Our study has highlighted that among the 23 selected variables in the final model, five could be related to diabetes “number of Glucose blood test”, “number of reimbursement of HbA1c tests”, “number of Fundus examination by biomicroscopy with contact lens”, “number of reimbursement of functional examination of the ocular motricity” and “number of reimbursement of binocular vision examination”. These tests/examinations could happen after a diagnosed diabetes (without treatment in our study) or also characterize a group with higher risk factors but with not diagnosed diabetes. Further, three variables among the five are not specific to diabetes (“number of Fundus examination by biomicroscopy with contact lens”, “number of reimbursement of functional examination of the ocular motricity” and “number of reimbursements of binocular vision examination. We also performed an additional analysis after implementing the model without these 5 variables, the accuracy was 65% (81% sensitivity and 65% specificity). In France, the screening recommendations for diabetes are based on the glucose blood test. HbA1c is only recommended for the management of diabetes but not for diagnoses. In 2009 and 2010, the WHO has introduced HbA1c as an alternative method to diagnose diabetes that has been adopted by many countries since this date. The ophthalmologic problems such as glaucoma, cataract, ocular movement disorders, etc., are the main complications of diabetes. Therefore, the increase frequency of medical acts performed as a result of diabetes related complications such as visual functions allowed to better characterize incident diabetes cases. Moreover, the increased use of non-diabetic drugs along with mentioned biological tests in incident diabetes group may explain potentially the pre-existing comorbidity or may be late diagnosis diabetes with cardiovascular or gastrointestinal diseases. The aim was to estimate the incidence of treated diabetes, therefore all participants with prior antidiabetic drugs (12–36 months before inclusion) were excluded and therefore the antidiabetic drugs were not included among 23 selected variables. The main reason to exclude these participants were to capture the true estimation of incidence of diabetes and took into account other variables to predict the incidence of diabetes.

### Implications and perspectives for future research

This innovative approach has been applied to two further studies: i. to classify and to estimate the prevalence of type 1 and type 2 diabetes cases [21] and, ii. to identify the number of undiagnosed diabetes cases ML algorithms in the SNDS (on going). For the first study, ML-algorithm developed has a sensitivity of 100% and specificity of 97%, and for the second study, the sensitivity is 71% and specificity is 61%.

The next step is to apply this algorithm on SNDS to estimate the incidence of type 2 diabetes cases. We recommend further research for following perspectives using ML-techniques: *first* to use different time windows (for example 6 months, 12 months or 16 months) to code variables and to explore their impact on estimates, *second* to predict the trend of diabetes over time and *third,* to estimate the contribution of determinants of diabetes such as BMI, dietary habits and physical activity, on developing type 2 diabetes using ML approaches.

## Conclusions

The use of MLT to analyze large administrative databases (health and non-health related data sources) is increasing across European countries in order to improve the public health surveillance and health policy process. Supervised machine learning is an innovative methodology for the development of algorithms to exploit large health administrative databases. It was the first step that we have developed a generic ML-algorithm with a moderate performance to estimate the incidence of diabetes in a training data set. The results of this study have highlighted important methodological steps to apply MLTs and their implications on large health administrative databases. The next step is to apply this algorithm on SNDS to estimate the incidence of type 2 diabetes cases. More research is needed to apply various MLTs to estimate the incidence of various health conditions and to calculate the contribution of various risk factors on developing type 2 diabetes.

## Supplementary Information

**Additional file 1.** It describes the summary of results with three boosted algorithms [boosted version of logistic regression, boosted C5.0 classification model, and XGBoost] to our set of four models [i.e., 1. Linear discriminant analysis (LDA), 2. Logistic regression (LR), 3. Flexible discriminant analysis (FDA) and 4. Decision tree model (C5)]. It includes Table S 1.1 (main analysis), Table S1.2 (sensitivity analysis: over sampling), Table S1.3 (sensitivity analysis: down sampling), Table S2 (description of models used and the set of hyperparameters explored) and Table S3 (Area under curve: AUC).

## Data Availability

Not applicable.

## Abbreviations

ML: Machine Learning
SNDS: Système National de Données Santé: French National Health Database
CONSTANCES: A population-based epidemiological cohort
AI: Artificial Intelligence
InfAct: Information for Action i.e., a joint action of Member States to establish a sustainable European health information system
WP: Work Package
CNAMTS: Caisse Nationale de l’Assurance Maladies des Travailleurs Salaries
SAQ: Self-administered Questionnaire
MQ: Medical Questionnaire
ATC: Anatomical Therapeutic Code
LDA: Linear Discriminant Analysis
LR: Logistic Regression
FDA: Flexible Discriminant Analysis
C5: Decision Tree
AUC: Area under Receiver Operating Characteristics Curve
Pos Pred Value: Positive Predictive Value
Neg Pred Value: Negative Predictive Value
95%CI: 95% Confidence Interval
BMI: Body Mass Index
WHO: World Health Organization
