# Supplementary material for: Use of artificial intelligence for public health surveillance: a case study to develop a machine Learning-algorithm to estimate the incidence of diabetes mellitus in France
Source: Arch Public Health. 2021 Sep 22;79:168. doi: 10.1186/s13690-021-00687-0 (PMC8456679; doi:10.1186/s13690-021-00687-0)
Supplement: Supplementary file 1 — Additional file 1. It describes the summary of results with three boosted algorithms [boosted version of logistic regression, boosted C5.0 classification model, and XGBoost] to our set of four models [i.e., 1. Linear discriminant analysis (LDA), 2. Logistic regression (LR), 3. Flexible discriminant analysis (FDA) and 4. Decision tree model (C5)]. It includes Table S 1.1 (main analysis), Table S1.2 (sensitivity analysis: over sampling), Table S1.3 (sensitivity analysis: down sampling), Table S2 (description of models used and the set of hyperparameters explored) and Table S3 (Area under curve: AUC). [file 13690_2021_687_MOESM1_ESM.docx]

**Additional file 1**

We have included three boosted algorithms [boosted version of logistic regression, boosted C5.0 classification model, and XGBoost] to our set of four models [i.e., 1. Linear discriminant analysis (LDA), 2. Logistic regression (LR), 3. Flexible discriminant analysis (FDA) and 4. Decision tree model (C5)].

We performed a sensitivity analysis with over and down sampling approaches including all models to analyze the predictive power of these approaches in the test data set.

The main analysis includes 23 variables and the sensitivity analysis includes 182. Due to the over sampling, a higher number of variables (i.e., 182) were selected leading to more complex model with better accuracy but more difficult to interpret in practice. We keep these same 182 variables for the second sensitivity analysis with a down-sampling procedure to have comparable results.

The result of this sensitivity analysis was significantly different from the already included algorithms. It increases the accuracy of the models with high number of variables.

**Table S1.1 : Main analysis (N = 23 variables)**

|  | **LDA** | **LR** | **FDA** | **C5** | **Boost_logreg** | **Boost_C5** | **XGBoost** |
| --- | --- | --- | --- | --- | --- | --- | --- |
| Accuracy : | 0.67 | 0.65 | 0.77 | 0.66 | 0.64 | 0.67 | 0.69 |
| 95% CI : | (0.6635, 0.6831) | (0.6401, 0.66) | (0.7614, 0.7789) | (0.6510, 0.6708) | (0.6300, 0.6500) | (0.6591, 0.6788) | (0.6797, 0.6990) |
| No Information Rate : | 0.998 | 0.998 | 0.998 | 0.998 | 0.998 | 0.998 | 0.998 |
| P-Value [Acc > NIR] : | 1 | 1 | 1 | 1 | 1 | 1 | 1 |
| Kappa | 0.0033 | 0.0041 | 0.0032 | 0.0024 | 0.0026 | 0.0052 | 0.0036 |
| McNemar's Test P-Value | <2e-16 | <2e-16 | <2e-16 | <2e-16 | <2e-16 | <2e-16 | <2e-16 |
| Sensitivity | 0.625 | 0.750 | 0.438 | 0.563 | 0.625 | 0.813 | 0.625 |
| Specificity | 0.673 | 0.650 | 0.771 | 0.661 | 0.640 | 0.669 | 0.690 |
| Pos Pred Value | 0.003 | 0.004 | 0.003 | 0.003 | 0.003 | 0.004 | 0.004 |
| Neg Pred Value | 0.999 | 0.999 | 0.999 | 0.999 | 0.999 | 0.999 | 0.999 |
| Detection Rate | 0.327 | 0.351 | 0.230 | 0.339 | 0.360 | 0.332 | 0.311 |
| Balanced Accuracy | 0.649 | 0.700 | 0.604 | 0.612 | 0.633 | 0.741 | 0.657 |

**Table S1.2 : Sensitivity analysis (over sampling) (N = 182 variables)**

|  | **LDA** | **LR** | **FDA** | **C5** | **Boost_logreg** | **Boost_C5** | **XGBoost** |
| --- | --- | --- | --- | --- | --- | --- | --- |
| Accuracy : | 0.91 | 0.93 | 0.65 | 0.99 | 0.92 | 0.99 | 0.82 |
| 95% CI : | (0.9018, 0.9139) | (0.9264, 0.937) | (0.6453, 0.6651) | (0.9884, 0.9925) | (0.9158, 0.9292) | (0.9865, 0.991) | (0.8114, 0.8274) |
| No Information Rate : | 0.998 | 0.998 | 0.998 | 0.998 | 0.998 | 0.998 | 0.998 |
| P-Value [Acc > NIR] : | 1 | 1 | 1 | 1 | 1 | 1 | 1 |
| Kappa | 0.0085 | 0.0063 | 0.0055 | 0.0204 | 0.0175 | 0.0168 | 0.0063 |
| McNemar's Test P-Value | <2e-16 | <2e-16 | <2e-16 | 7.348e-09 | <2e-16 | 8.243e-12 | <2e-16 |
| Sensitivity | 0.312 | 0.187 | 0.875 | 0.062 | 0.555 | 0.062 | 0.500 |
| Specificity | 0.909 | 0.933 | 0.654 | 0.992 | 0.923 | 0.990 | 0.820 |
| Pos Pred Value | 0.006 | 0.005 | 0.004 | 0.014 | 0.010 | 0.011 | 0.005 |
| Neg Pred Value | 0.998 | 0.998 | 0.999 | 0.998 | 0.999 | 0.998 | 0.998 |
| Detection Rate | 0.000 | 0.000 | 0.001 | 0.000 | 0.000 | 0.000 | 0.001 |
| Balanced Accuracy | 0.610 | 0.560 | 0.764 | 0.527 | 0.739 | 0.526 | 0.660 |

**Table S1.3 : Sensitivity analysis (down sampling) (N = 182 variables)**

|  | **LDA** | **LR** | **FDA** | **C5** | **Boost_logreg** | **Boost_C5** | **XGBoost** |
| --- | --- | --- | --- | --- | --- | --- | --- |
| Accuracy : | 0.66 | 0.51 | 0.75 | 0.61 | 0.75 | 0.74 | 0.68 |
| 95% CI : | (0.6548, 0.6744) | (0.5046, 0.5255) | (0.746, 0.7639) | (0.6015, 0.6218) | (0.7433, 0.7614) | (0.7361, 0.7543) | (0.6723, 0.6918) |
| No Information Rate : | 0.998 | 0.998 | 0.998 | 0.998 | 0.998 | 0.998 | 0.998 |
| P-Value [Acc > NIR] : | 1 | 1 | 1 | 1 | 1 | 1 | 1 |
| Kappa | 4e-04 | 0.0015 | 0.003 | 0.003 | 0.004 | 0.004 | 0.0041 |
| McNemar's Test P-Value | <2e-16 | <2e-16 | <2e-16 | <2e-16 | <2e-16 | <2e-16 | <2e-16 |
| Sensitivity | 0.375 | 0.687 | 0.500 | 0.812 | 0.562 | 0.562 | 0.687 |
| Specificity | 0.665 | 0.514 | 0.755 | 0.611 | 0.752 | 0.745 | 0.682 |
| Pos Pred Value | 0.002 | 0.002 | 0.003 | 0.003 | 0.004 | 0.003 | 0.003 |
| Neg Pred Value | 0.998 | 0.998 | 0.998 | 0.999 | 0.998 | 0.998 | 0.999 |
| Detection Rate | 0.000 | 0.001 | 0.001 | 0.001 | 0.001 | 0.00& | 0.001 |
| Balanced Accuracy | 0.520 | 0.601 | 0.627 | 0.711 | 0.657 | 0.654 | 0.684 |

**Table S2: The models used and the set of hyperparameters explored**

| **Model** | **Hyperparameter space explored** |
| --- | --- |
| *Logistic regression* | Not applicable |
| *Boosted logistic regression* | Number of iterations: 1, 6, 11, …, 101 |
| *Single C5.0 Tree* | Not applicable |
| *Boosted C5.0* | Model: tree or ruleset  Number of iterations: 1, 6, 11, … 96 |
| *Linear Discriminant Analysis* | Not applicable |
| *Flexible Discriminant Analysis* | Random combinations (30) of product degree and number of terms. |
| *XGBoost* | Booster: tree  Minimum loss reduction (gamma): 0  Minimum sum of instance weight needed in a child: 1  Step shrinkage (eta): 0.1, 0.01  Maximum depth: 2, 4, 6  Number of iterations: 1, 21, 41, …, 981 |

**Table S3: Area under curve (AUC)**

|  | **Main analysis** | **Sensitivity analysis (over sampling)** | **Sensitivity analysis (down sampling)** |
| --- | --- | --- | --- |
| **LDA** | 0.7226 | 0.6556 | 0.6045 |
|  | 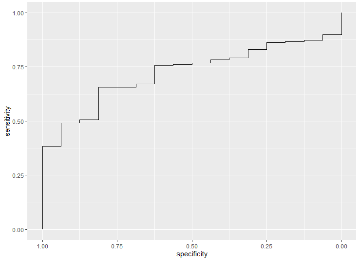 | 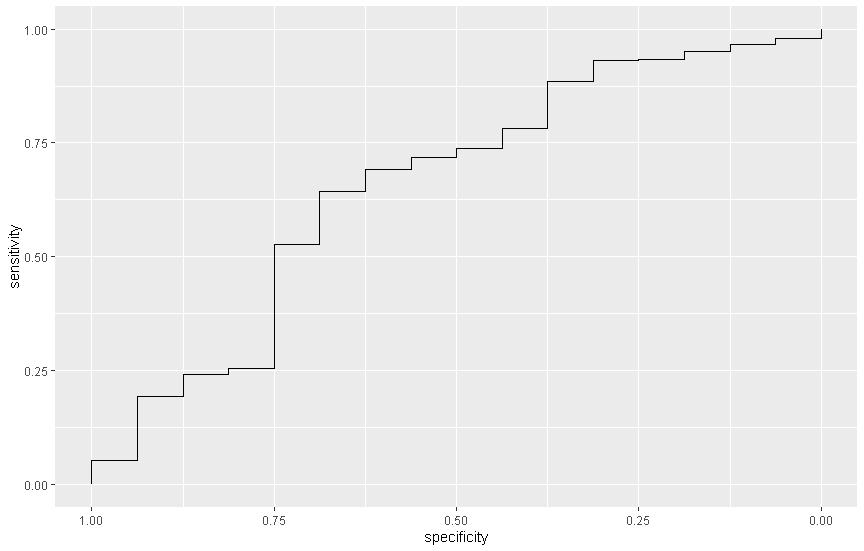 | 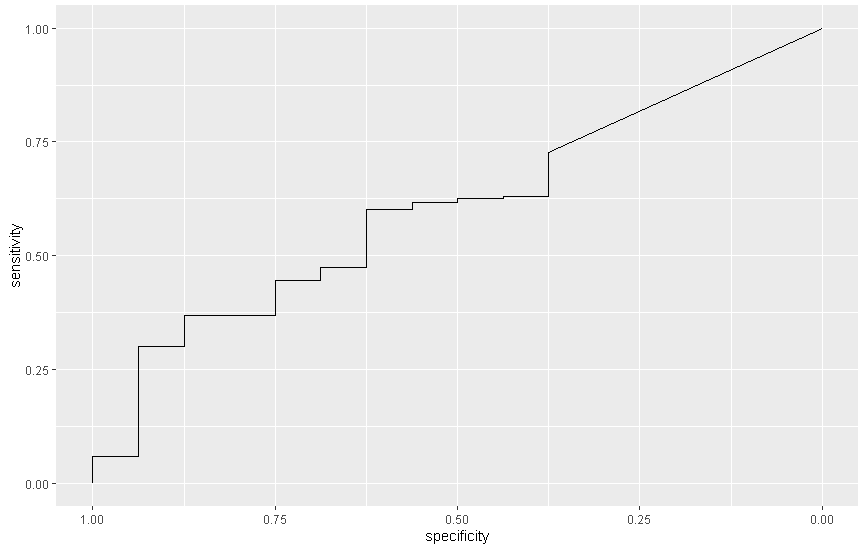 |
| **LR** | 0.7208 | 0.5989 | 0.6085 |
|  | 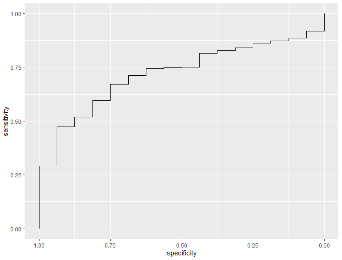 | 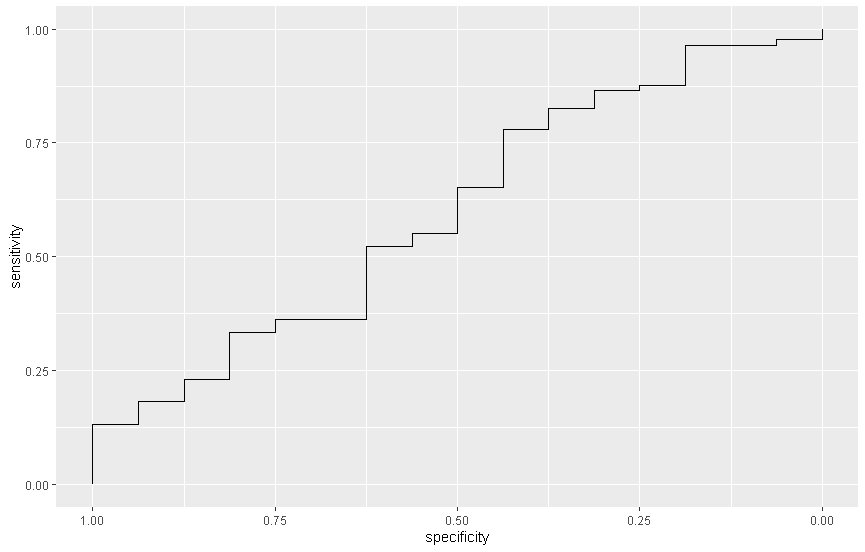 | 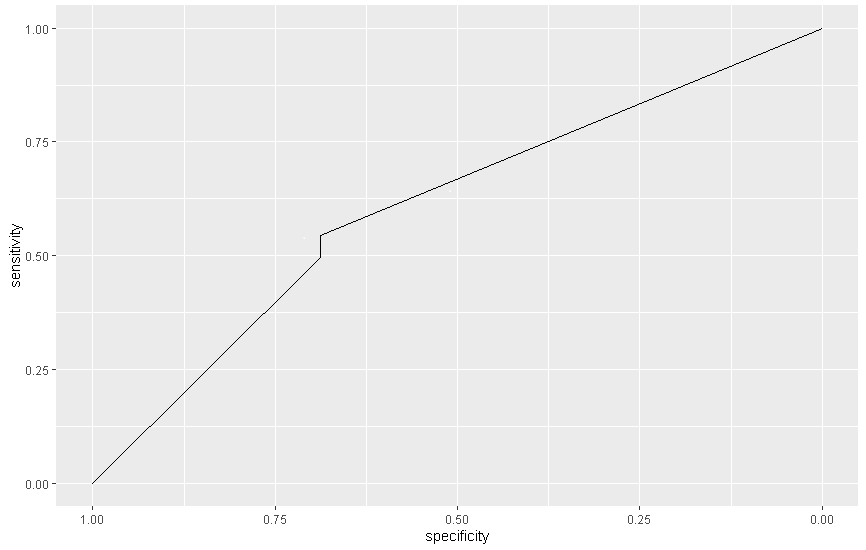 |
| **FDA** | 0.6286 | 0.8031 | 0.6706 |
|  | 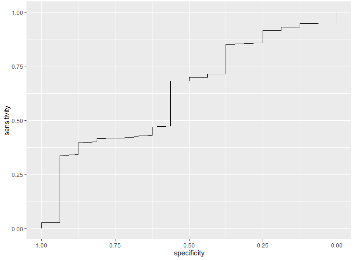 | 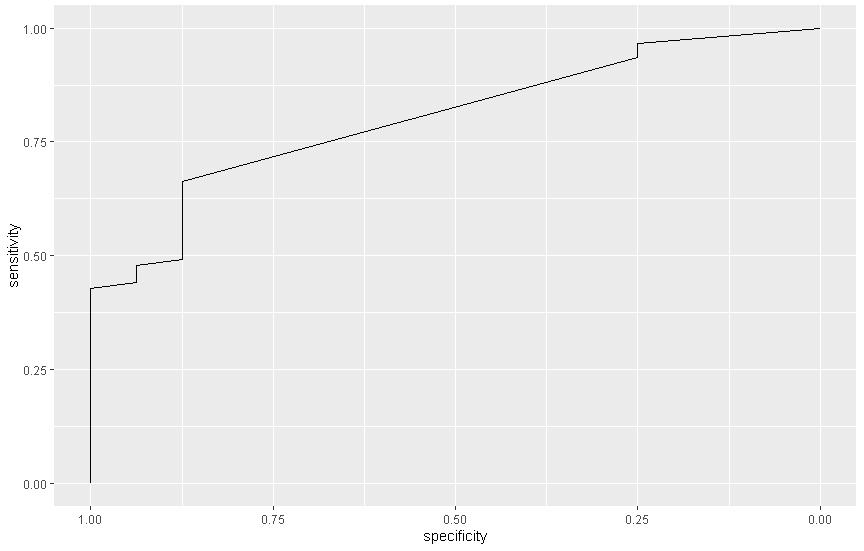 | 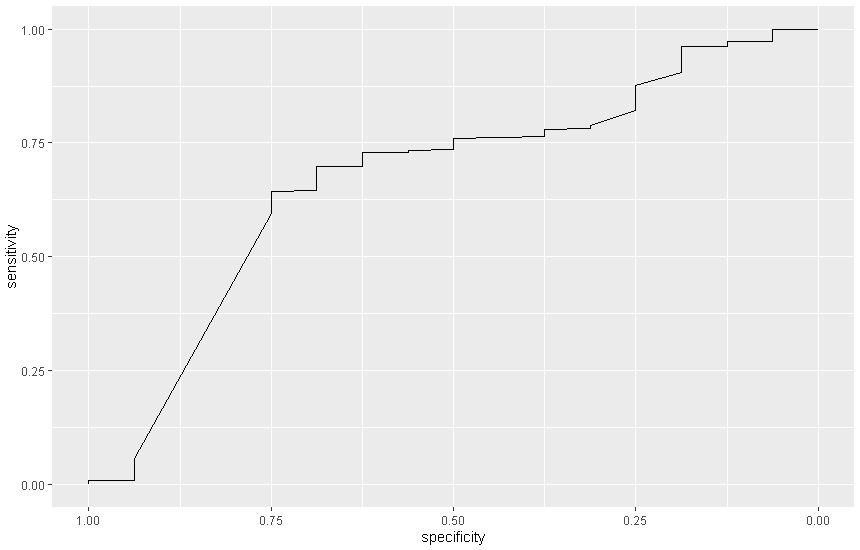 |
| **C5** | 0.6238 | 0.6611 | 0.7266 |
|  | 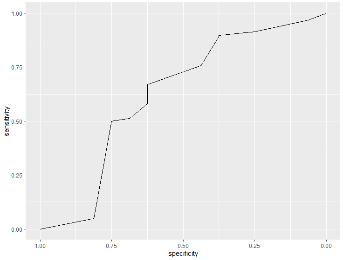 | 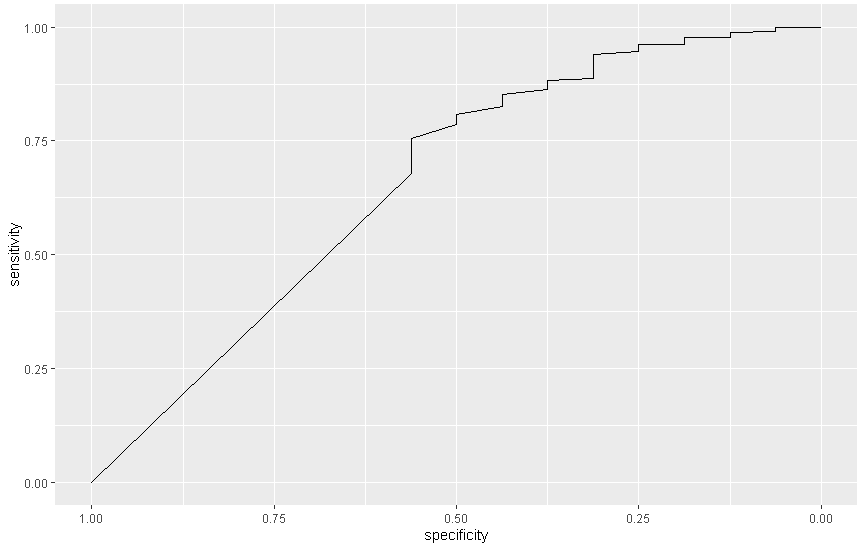 | 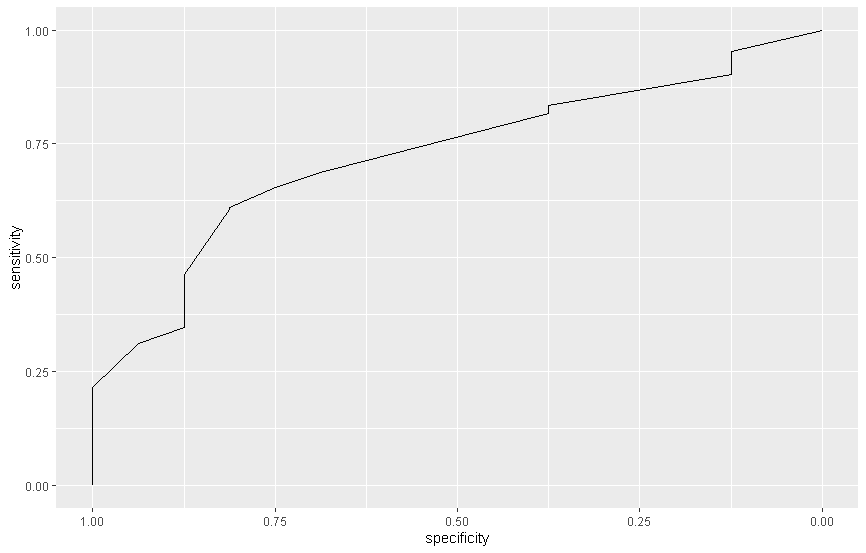 |
| **Boost_logreg** | 0.4107 | 0.7354 | 0.7282 |
|  | 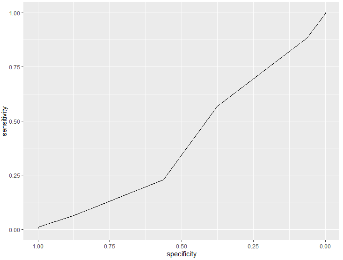 | 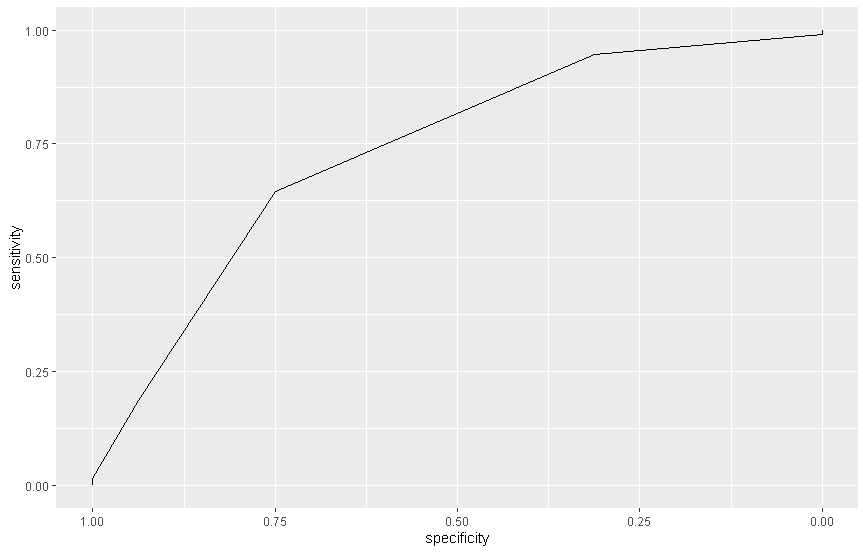 | 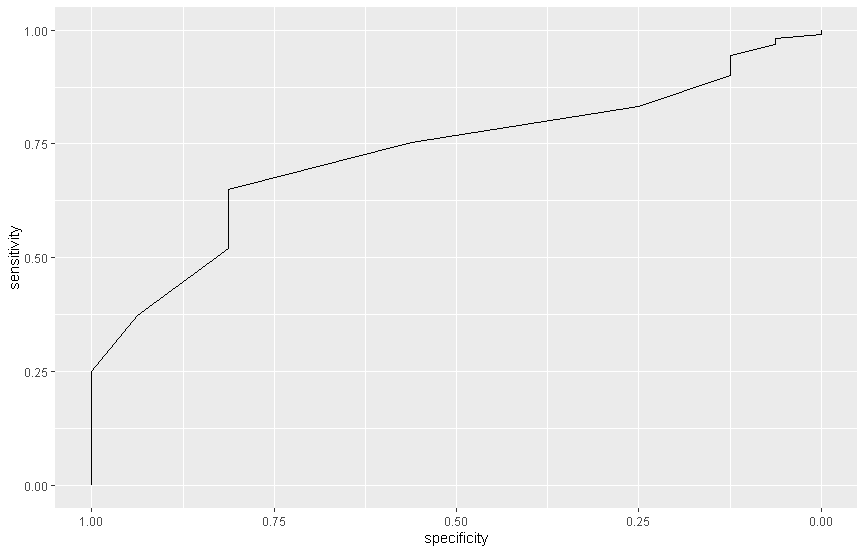 |
| **Boost_C5** | 0.7136 | 0.6618 | 0.6907 |
|  | 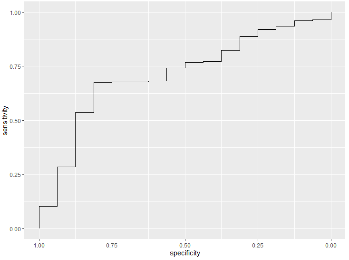 | 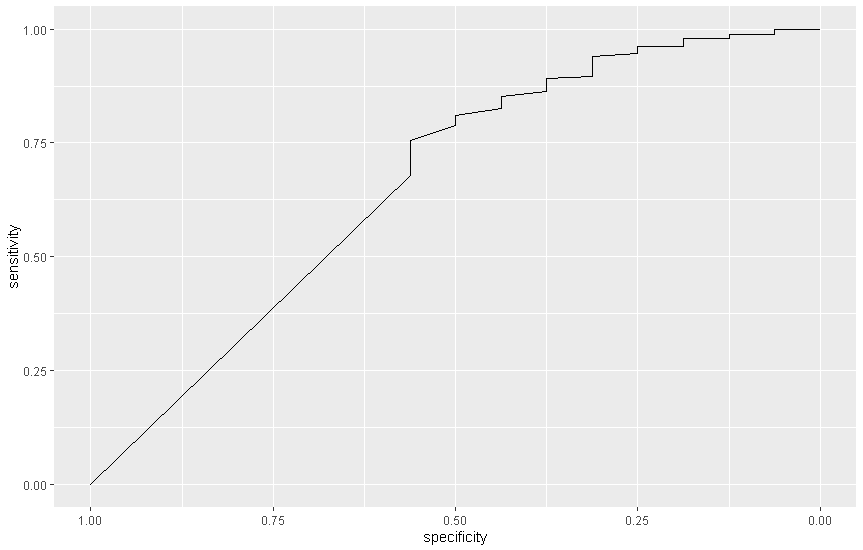 | 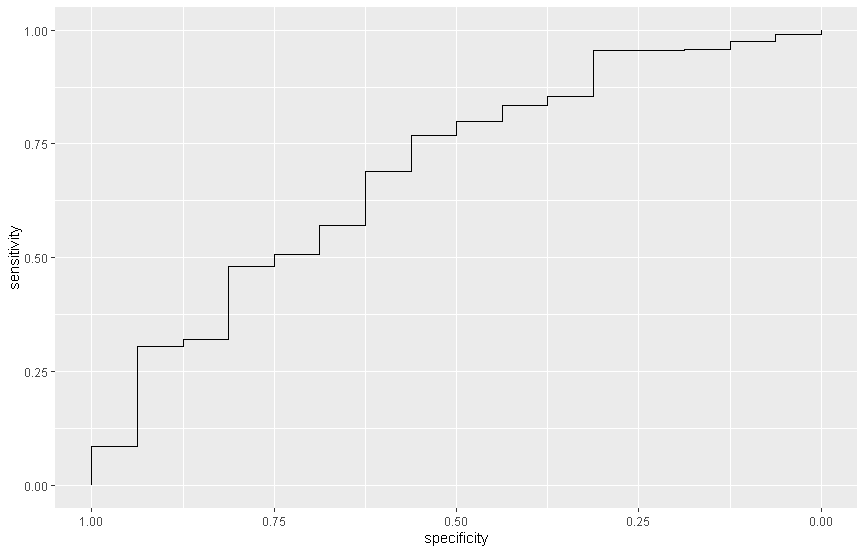 |
| **XGBoost** | 0.732 | 0.7282 | 0.7277 |
|  | 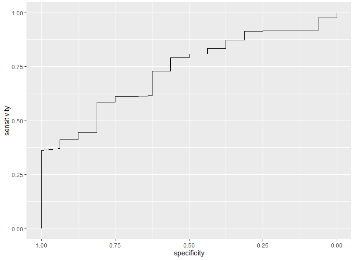 | 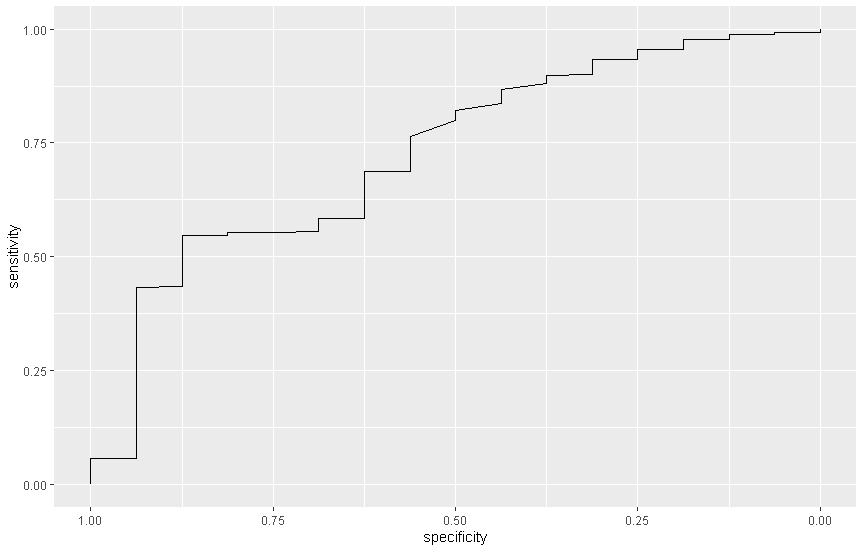 | 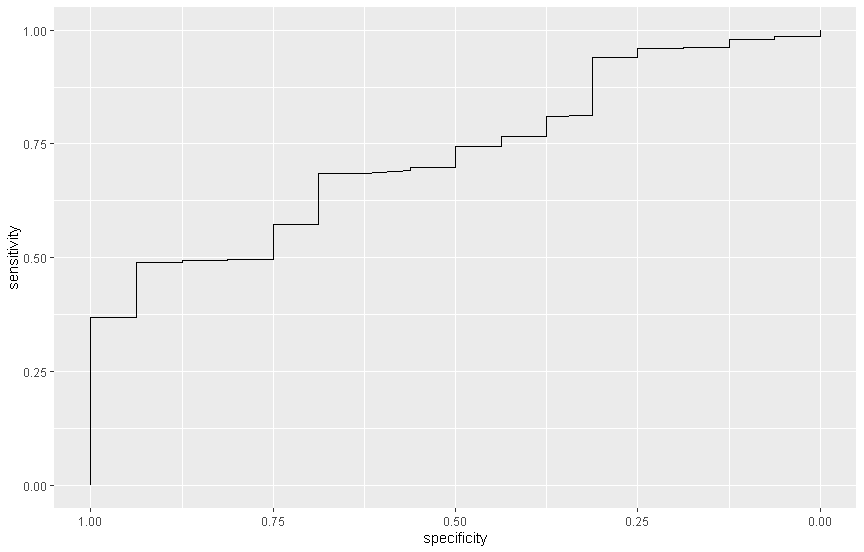 |
